# Sex differences in mortality of admitted patients with personality disorders in North Norway - a prospective register study

**DOI:** 10.1186/1471-244X-13-317

**Published:** 2013-11-26

**Authors:** Anne Høye, Bjarne K Jacobsen, Vidje Hansen

**Affiliations:** 1 Centre for Clinical Documentation and Evaluation (SKDE), University Hospital of North Norway (UNN), Mailbox 6, University Hospital of North Norway, 9037 Tromsø, Norway; 2 Institute of Clinical Medicine, Faculty of Health Sciences, University of Tromsø and University Hospital of North Norway, 9037 Tromsø, Norway; 3 Department of Community Medicine, Faculty of Health Sciencies, University of Tromsø, 9037 Tromsø, Norway

## Abstract

**Background:**

It is well established that patients with serious mental disorders have higher mortality than the general population, yet there are few studies on mortality of both natural and unnatural causes in patients with personality disorders. The aim of this study was to investigate the mortality of in-patients with personality disorder diagnosis in a 27-year follow-up cohort in North Norway, with a special focus on gender differences.

**Method:**

Based on a hospital case register covering 1980 to 2006, 284 female and 289 male patients were included. The cohort was linked to the Norwegian Cause of Death Registry for information concerning mortality. The mortality rates were adjusted for age by applying a Poisson regression model. The relative mortality in men compared to women was tested with Cox regression with attained age as the time variable. The number of deaths to be expected among the patients if the mortality rates of the general population in Norway had prevailed was estimated and excess mortality, expressed by the standardized mortality ratio (SMR), calculated.

**Results:**

When compared to the mortality in the general population, men and women with personality disorder diagnoses had 4.3 (95% CI: 3.2 - 5.9) and 2.9 (95% CI: 1.9 - 4.5) times, respectively, increased total mortality. Patients with personality disorder diagnoses have particularly high mortality for unnatural deaths; 9.7 (95% confidence interval (CI): 6.3 - 15.1) times higher for men and 17.8 (95% CI: 10.1 - 30.3) for women, respectively, and even higher for suicides – 15 (95% CI: 9–27) for men and 38 (95% CI: 20–70) for women. The mortality due to natural causes was not statistically significantly increased in women, whereas men had 2.8 (95% CI: 1.8 - 4.4) times higher mortality of natural deaths than the general population.

**Conclusion:**

Compared to the general population, patients with a personality disorder have high mortality, particularly mortality from unnatural causes. The number of deaths caused by suicides is especially high for women. Men also have higher mortality of natural causes than the general population.

## Background

Patients with mental disorders have higher mortality than the general population [1]. In Denmark, Sweden and Finland the mortality of patients who at some point have been admitted to hospital because of a mental disorder is shown to be two to three times higher than the general population [2]. Life expectancy for patients with personality disorder diagnoses is significantly reduced [3].

Wahlbeck et al. [2] show in a large register-based study from Sweden, Denmark and Finland that the life expectancy gap of 20 years for individuals with serious mental disorders compared to that in the general population is more or less unchanged, in spite of some progress in Denmark and Finland. In Norway, mortality of patients with mental illness has been reported infrequently since 1916 [4-6], but there have been no national studies on mortality for patients with mental disorders. Although there are some differences between the Nordic countries, there is an overall decline in suicide mortality from 1980 to 2009 [7]; from 25–50 to 20–36 per 100 000 for men, and from 9–26 to 8–11 per 100 000 for women. Suicide is, however, still the second most common cause of death in the age group 15–24 years, and there is an upward trend among young women in Norway and Finland. Furthermore, there are pronounced regional differences; in Norway, the annual rate for men at all ages was > 35 per 100 000 inhabitants in Finnmark, the northernmost county.

Patients with personality disorder diagnoses have high rates of unnatural deaths due to suicide, accidents and substance abuse [3,8,9]; the subgroup with the undoubtedly highest mortality being borderline personality disorder. The substantially increased total mortality in patients with personality disorders strongly suggests a higher mortality also due to natural causes [8,10], but this has been less investigated. Unstable personality traits are correlated with reduced physical health across the life span [11], and disordered personality is found to be significantly predictive of impaired physical functioning [12]. Certain personality characteristics such as high negative emotionality are associated with negative health outcomes linked to cardiovascular disease and diabetes [13]. Hiroeh et al. [10] found that the standardized mortality ratio (SMR) for all natural cause mortality was higher across a range of psychiatric illnesses in both sexes, including personality disorder diagnosis. The relative mortality compared with the general population (SMR) is higher in women than in men for homicide, suicide and accident in patients with personality disorder diagnoses [9].

The aims of the present study were to compute the excess mortality of men and women with personality disorder diagnoses admitted to psychiatric hospital in Northern Norway compared to the general population, and to document the differences in age-adjusted mortality rates and subgroup characteristics between male and female patients.

## Methods

The University Hospital of North Norway is the only psychiatric hospital covering the two northernmost counties in Norway; Troms and Finnmark. These counties comprise a vast area of approximately 75 000 km^2^, with a total of 234 952 inhabitants in 2012. Information about each admission has been collected since 1980, and this computerized register has been regularly checked for validity against patient files. The information used in this study was the unique 11-digit personal identification number assigned to every Norwegian resident; date of admission; commitment status; date of discharge and diagnoses at discharge. The present study includes all admissions from January 1st 1980 to December 31st 2006. During this period of 27 years there was a stable bed-to-population ratio of approximately 4 per 10 000 inhabitants.

The personal identification number was used for linkage of the register with the Norwegian Cause of Death Registry and the information concerning emigration held by Statistics Norway. The Cause of Death Registry covers data on all persons with residence in the country, independent of whether they died in Norway or not.

In the study period there were 22434 admissions; 5840 persons were admitted, 3116 men and 2724 women. A total of 573 patients were diagnosed with a personality disorder at discharge on at least one of their admissions, 289 (50.4%) of them were men. Personality disorder was defined according to the International Classification of Diseases [14,15] (ICD-9: 301.0 - 301.9, ICD-10: F60-F69). Before 1985, ICD-8 was used, and the diagnoses in this period were converted retrospectively to ICD-9 codes.

The 573 patients were observed from the date of first admission after January 1st 1980 until death, moving abroad (10 subjects) or the end of 2006, for a total of 6624 person years. Fourteen subjects who were in-hospital patients at January 1st 1980 were included in the cohort, with start of follow-up the same day. In Norway, all death certificates are completed by a physician and coded in accordance with the current ICD-system. The diagnoses recorded by the Cause of Death Registry as the underlying cause of death were used.

### Statistical analyses

Differences in the characteristics of male and female patients (Table 1) were tested with t-test, Wilcoxon rank sum test (when the distributions were markedly positively skewed) or chi-square test. We studied the differences in age-adjusted mortality rates between men and women with a personality disorder in the study cohort, and sex differences in standardized mortality ratios (SMR) compared to the general population.

**Table 1:** Characteristics of the patients with personality disorders according to gender

	**Men**	**Women**	p-value*
Number of patients	289	284	
Number of deaths	41	21	
Number of admissions	1924	3139	
Total time of follow-up (person-years)	3420	3204	
Follow-up time in years (mean, SD)	11.8 (7.4)	11.3 (7.4)	0.37
Age at first admission in years (mean, SD)	31.2 (9.9)	30.2(12.2)	0.28
Total number of days admitted (median, 1 and 3 quartile)	70 (18.0, 214.5)	111 (25.3, 293.0)	0.013
Number of admissions (median, 1 and 3 quartile)	3 (1,8)	4 (2,1)	0.004
% with personality disorder as only diagnosis (N)	30.1 (89)	23.9 (68)	0.07

*Difference between men and women.

Age adjustments of the mortality rates were carried out applying a Poisson regression model including gender, person-years, age (1-year interval) and number of deaths in the analyses. The relative mortality for men compared to women was tested with Cox regression with attained age as the time variable. For comparison with the mortality of the general population of Norway, indirect age adjustment was used. The number of deaths to be expected among the patients, if the mortality rates of the general population in Norway according to age (5-year groups) and calendar year (5-year groups) during follow-up had prevailed, were calculated. The ratio of the observed to the expected numbers of deaths, the standardized mortality ratio (SMR), expresses the relative mortality of the patient group compared to that of the general population. Confidence intervals for the SMRs were computed.

P-values < 0.05 were considered statistically significant. Statistical analyses were performed with SAS 9.2.

## Results

Table 1 gives the characteristics of the admissions. The cohort includes 289 men and 284 women who were diagnosed with a personality disorder during the study period. These 573 patients had a total of 5063 admissions, 1924 of them by men and 3139 of them by women. The mean age at first admission was 30.7 years, and they were followed by a mean of 11.6 years. Twenty-seven percent, 89 men and 68 women, had personality disorder as their only diagnosis, although they may have had several admissions. The proportion with personality disorder as the only diagnosis did not differ much between men (30.1% of the patients) and women (23.9%) (p = 0.07), but if any additional diagnosis, women tended to get mood disorder diagnoses whereas men got alcohol and substance abuse disorders diagnoses (results not shown).

The distribution of diagnostic subtypes of personality disorders was different in men and women (p < 0.001) (Table 2) as 30.1% and 52.8% of the male and female patients, respectively, had an emotionally unstable personality disorder diagnosis. Dissocial personality disorder and schizoid personality disorder, on the other hand, were more frequently found in men.

**Table 2:** Percentage distribution of personality disorder subtypes in men and women

**ICD subtypes**	**Men (n = 289)**	**Women (n = 284)**	**Total (n = 573)**	p-value*
Paranoid (F 60.0)	10.0	6.7	8.4	< 0.001
Schizoid (F60.1)	16.3	6.0	11.2	
Dissocial (F 60.2)	8.0	0.4	4.2	
Emotionally unstable (F 60.3)	30.1	52.8	41.4	
Anankastic (F60.5), Anxious (F60.6), Dependent (F 60.7)	8.0	10.9	9.4	
Mixed	27.7	23.2	25.5	

*Difference between men and women.

### Mortality in the cohort

A total of 41 men and 21 women died during 1980–2006 (Table 3). Twenty of the 41 deaths in men and 12 of the 21 deaths in women were due to unnatural causes. Twenty-two of the 32 unnatural deaths were suicides.

**Table 3:** Standardized mortality ratio for men and women in patients with personality disorder diagnosis

	**Men**			**Women**		
	Number of deaths	Expected number of deaths	SMR	Number of deaths	Expected number of deaths	SMR
Total mortality	41	9.46	4.3 (3.2 - 5.9)	21	7.13	2.9 (1.9 - 4.5)
Natural causes	21	7.40	2.8 (1.8 - 4.4)	9	6.45	1.4 (0.7 - 2.7)
Unnatural causes	20	2.06	9.7 (6.3 - 15.1)	12	0.67	17.8 (10.1 - 30.3)
*Suicide*	12	0.79	15 (9 - 27)	10	0.26	38 (20 - 70)

Figures are SMR (95% confidence interval).

The age-adjusted mortality rates were 12.4 and 6.1 per 1000 person-years in men and women, respectively (p = 0.01). Thus, men had twice the mortality rate compared to women; the age-adjusted hazard ratio (HR) was 2.0 (95% CI: 1.2 - 3.5). Statistically significantly higher mortality in men than in women was found for natural deaths (HR: 3.0, 95% CI: 1.3 - 7.0), but not for unnatural deaths (HR: 1.4, 95% CI: 0.7 - 3.0) or suicides (HR: 1.1, 95% CI: 0.5 - 2.5 based on 12 and 10 suicides in men and women, respectively) (results not shown in table).

In a sub-analysis, we restricted the cohort to the 157 patients, 89 men and 68 women, who did not receive any other diagnoses than personality disorder during their stays. There were 20 deaths during follow up, 15 in men and 5 in women. This analysis confirmed a higher total mortality in men than in women also in this group of patients (HR: 2.7, 95% CI: 0.9 – 8.5).

### Mortality in patients with a personality disorder compared to the general population (SMR)

Men and women had 4 and 3 times, respectively, increased mortality compared to the general population (Table 3); SMRs were 4.3 (95% CI: 3.2 - 5.9) for men and 2.9 (95% CI: 1.9 - 4.5) for women. The increased SMR for natural causes in women was not statistically significant (SMR 1.4 (95% CI: 0.7 - 2.7 based on 9 deaths), whereas the opposite was true for men; SMR was 2.8 (95% CI: 1.8 - 4.4), based on 21 deaths. The SMRs for unnatural deaths were particularly high both for men and women; 9.7 (95% CI: 6.3 - 15.1) and 17.8 (95% CI: 10.1 - 30.3), respectively, and even higher for suicides: 15 (95% CI: 9–27, based on 12 deaths) in men and 38 (95% CI: 20–70, based on 10 deaths) in women. Thus, for all-cause and natural causes mortality, the SMRs were higher in men than in women whereas the opposite was true for unnatural causes (Table 3). However, the sex differences were statistically significant only for suicides (p = 0.03).

We also note that the SMRs for total mortality were higher for subjects when aged < 50 (5.2 (95% CI: 3.5 - 7.8), based on 23 deaths in men and 5.8 (95% CI: 3.2 - 10.5), based on 11 deaths in women) than when aged 50 and above (3.6 (95% CI: 2.4 - 5.7), based on 18 deaths in men and 1.9 (95% CI: 1.0 - 3.5), based on 10 deaths in women).

In the group of 157 patients who did not receive any other diagnoses than personality disorder, a statistically significantly increased SMR for total mortality was found in men (SMR: 3.5, 95% CI: 2.1, 5.8), but not in women (SMR: 1.6, 95% CI: 0.7, 3.8). The latter estimate was based on 5 deaths, though. Separate analysis restricted to patients with at least one diagnosis of emotionally unstable personality disorder (with 18 deaths in men and 10 in women) demonstrated even higher SMR estimates for total mortality (6.6 (95% CI: 4.1, 10.4) in men and 5.5 (95% CI: 3.0, 10.3) in women) than those presented in Table 3. SMRs for suicides were 17 (95% CI: 7, 42) and 40 (95% CI: 18, 88) in men and women, respectively.

## Discussion

### Mortality

Our finding of 3–4 times higher all-cause mortality of patients with personality disorder diagnoses than in the general population is in accordance with other studies [3,8]. Mortality for natural causes was not statistically significantly higher for women compared to the general female population, while men had an almost threefold increased risk of dying from natural causes. This is in variance with the findings from Denmark [10], where there were increased SMRs for both men and women. Our study shows that for both men and women, the rates of death of unnatural causes are very high compared to the rates in the general population, with SMR for suicide being especially high for women. Hiroeh [9] found 12 and 16 times higher suicide mortality in male and female patients with personality disorder diagnoses, respectively, while our study shows an SMR of 15 for men and 38 for women. In a Norwegian follow-up study of mortality after hospital-treated self-poisoning, SMR for suicide was 17.8 for men and 46.4 for women [16]. This is comparable to our results.

The higher mortality could be partly explained by selection due to admission practice, with a strong focus on decentralized psychiatric health services as well as very long travelling distances to the psychiatric hospital in Northern Norway. These factors may have led to a high threshold for admitting patients. Community studies conducted over the past decade estimate that 6–10% of individuals in the general population fulfill criteria of a personality disorder [17]. The patients admitted to the hospital are by no means a random sample of them. The median number of admissions was 4, and the median total length of the admissions was 84 days. Approximately two out of three were at some point involuntarily committed (results not shown).

Patient selection with regard to diagnosis may also be of importance. In clinical populations, borderline personality disorder is the most common personality disorder, with a prevalence of between 15 – 25% of all in-patients [3]. ICD-10 estimates of any personality disorder are lower than estimates based on DSM-IV, but the difference is smaller for borderline/emotionally unstable personality disorder than for other subtypes [17]. In our total cohort of 5840 inpatients only 4.7% are diagnosed with emotionally unstable personality disorder. The higher mortality may therefore indicate a strong association with symptom severity, and admissions may be more extensively triggered by serious suicidal threats or attempts [18]. Symptom severity may also be reflected in less social and family support, leading to admission as the only solution in a crisis.

The SMRs are based on the entire Norwegian population. Northern Norway had slightly higher mortality rates for men than the rest of Norway during most of the follow-up period [19], and this might have led to slightly higher SMRs for men in our study. Comparing to the population in North Norway would, however, have led to biased (low) SMRs because the deaths of patients in the cohort contribute particularly to the total number of unnatural deaths in the population of North Norway.

More than 40% of the 573 patients with personality disorder diagnoses had borderline or emotionally unstable personality disorder (ICD-9 301.83, ICD-10 F60.3). Unnatural deaths due to suicide, accidents and substance abuse strongly contribute to the higher mortality of the total group of patients with personality disorders [3]. About 10% of all patients with borderline personality disorder diagnosis commit suicide [20,21], with higher rates in studies with longer follow-up [22]. Multiple suicide attempts and life-threatening self-harm behaviors are common [23] as 60 – 70% of the individuals with borderline personality disorder diagnosis attempt suicide during the course of their illness [24]. Even if the high level of suicide threats, attempts and self-injury is often seen in younger patients, the mean age of suicide completers is found to be as low as 30–37 years [8,20-22].

The main sex differences seen in our study are the higher SMRs for natural causes in men than women, and the very high mortality for suicide in women. These differences seem to be closely correlated with the distribution of personality disorder subtypes as shown in Table 2. Admitted men were diagnosed with dissocial and schizoid personality disorder significantly more often than women, and it is likely that these personality traits lead to underreporting of somatic symptoms and less help seeking, contributing to the higher mortality for natural causes. There is no evidence that emotionally unstable personality disorder in general is more common in women [8]. Women are known to seek medical help to a higher degree than men [25]. This could lead to an overrepresentation of women in the patient population, but could also have attenuated the correlation between symptom severity and mortality in women.

Among the strengths of this study are the long follow-up time and the completeness of data concerning the admitted patients, with regular data control against patient files. Also, there is virtually no loss to follow-up as the Norwegian Cause of Death Registry must be considered complete with regard to mortality [26].

There are no private psychiatric hospitals in Norway. Being the only regional psychiatric hospital, all residents from the two counties admitted to any psychiatric hospital in Norway during the entire period will be transferred to the University Hospital of North Norway. The mental health services in the two counties are almost exclusively public, with well-established structures for cooperation on patient admission and follow-up. Hence, very few admissions of subjects who are residents in these two counties in Norway have taken place elsewhere without eventually being included in our database.

### Validity

All diagnoses are made by clinical consensus and not by standardized diagnostic procedures. Diagnoses are registered on the day of discharge by the treating clinician, usually derived by a team discussion. The proportion of admitted patients with a personality disorder diagnosis was quite stable during 1980–2006 (2–7% of patients admitted), but diagnostic practice and hence diagnostic reliability is not known. It is likely that this would have affected the validity of the diagnoses. The general construct validity of the personality disorder diagnoses has, however, been widely discussed [23,27,28]. Self-attributed personality traits seem to be more stable than symptoms assessed by clinicians, and diagnostic stability is generally found to be low [29]. Disease course is heterogeneous, and whether criteria for the diagnoses are met seems to vary depending on what is going on in the patients’ lives [3]. A high rate of comorbidity is well known in patients with personality disorders [3,8]. 85% of patients with borderline personality disorder meet criteria for having one or more 12-month axis I disorders according to DSM-IV [3]. The dimensional nature of personality disorders has long been argued, and it is claimed by many that the normal and pathological personality traits are continuous rather than categorical [30].

There are indications of high comorbidity rates also in our cohort, as only 27% had personality disorder as their only diagnosis, but since comorbidity seems to be underdiagnosed in psychiatric case registers at least in Norway [31], we cannot draw any firm conclusions. Still, it is likely that many of the admitted patients have additional alcohol and substance abuse, psychotic or serious affective symptoms, factors associated with higher mortality risk [3,32] and help seeking [33]. Hence, comorbidity may have increased SMRs in our study for both men and women. The high mortality and sex differences seen in a population of admitted patients with personality disorder diagnoses are clinically relevant, and should lead to caution and strengthening of follow-up after discharge with a stronger focus on differentiating between men and women.

## Conclusion

Our study confirms a significant mortality gap between patients with a personality disorder and the general Norwegian population, with a very high risk of committing suicide. The excess risk in women was particularly high; more women committed suicide than died of all natural causes during follow-up. Mortality for natural causes of death was not statistically significantly higher for women compared to the general population, while men had also an almost threefold increased risk of dying from natural causes than the general male population.

### Ethical standards

The study was approved by The Regional Committee for Medical and Health Research Ethics. The approval includes exemption of written consent, which according to Norwegian legislation can apply to research that uses information collected routinely in health care services as long as the participants’ welfare and integrity are maintained.

## Competing interests

The authors declare that they have no competing interests.

## Authors’ contributions

AH has the main responsibility for drafting and revisions of the manuscript. BKJ contributed to design of the study, data analysis and critical revision of the manuscript, VH contributed to design of the study and critical revision of the manuscript. All authors have read and approved the final manuscript.

## Pre-publication history

The pre-publication history for this paper can be accessed here:

http://www.biomedcentral.com/1471-244X/13/317/prepub
